# Teaching Quality in Math Class: The Development of a Scale and the Analysis of Its Relationship with Engagement and Achievement

**DOI:** 10.3389/fpsyg.2017.00895

**Published:** 2017-06-28

**Authors:** Jaime Leon, Elena Medina-Garrido, Juan L. Núñez

**Affiliations:** Faculty of Educational Sciences, University of Las Palmas de Gran CanariaLas Palmas, Spain

**Keywords:** teacher behavior/beliefs, mathematics, motivation, student engagement, education assessment

## Abstract

Math achievement and engagement declines in secondary education; therefore, educators are faced with the challenge of engaging students to avoid school failure. Within self-determination theory, we address the need to assess comprehensively student perceptions of teaching quality that predict engagement and achievement. In study one we tested, in a sample of 548 high school students, a preliminary version of a scale to assess nine factors: teaching for relevance, acknowledge negative feelings, participation encouragement, controlling language, optimal challenge, focus on the process, class structure, positive feedback, and caring. In the second study, we analyzed the scale’s reliability and validity in a sample of 1555 high school students. The scale showed evidence of reliability, and with regard to criterion validity, at the classroom level, teaching quality was a predictor of behavioral engagement, and higher grades were observed in classes where students, as a whole, displayed more behavioral engagement. At the within level, behavioral engagement was associated with achievement. We not only provide a reliable and valid method to assess teaching quality, but also a method to design interventions, these could be designed based on the scale items to encourage students to persist and display more engagement on school duties, which in turn bolsters student achievement.

## Introduction

Unlock students’ academic potential is a priority for many researchers and practitioners within the educational context (Hulleman et al., 2016). Academic failure has consequences not only during adolescence, when low academic performance results in feelings of failure and eventually to drop out (Valiente et al., 2014), but also in the future, as adults who did not complete their studies are more likely to have health problems and to need social services (Levpuscek et al., 2012; Blankson and Blair, 2016). Within school subjects, mathematics plays a fundamental role for its implication in other school subjects (Gaspard et al., 2015), importance in future social and labor success (Seaton et al., 2014), effects on decisions making in a changing and ambiguous society (Meder and Gigerenzer, 2014), and its relationship with the Gross Domestic Product (OECD, 2010).

Unfortunately, student math achievement and engagement declines meaningfully all the way through secondary education (Kiemer et al., 2015; Stroet et al., 2015b). Thus, educators at this developmental stage are faced with the challenge of engaging students to learn and achieve. To address this issue, researchers guided by self-determination theory (SDT; Deci and Ryan, 1985, 2000), a broad framework for the study and explanation of human motivation and personality (Liu et al., 2016b), have shown evidence of the role played by the teaching quality (for an overview see: Ryan and Deci, 2009; Núñez and León, 2015). However, knowledge in defining the precise components that lead to an optimal functioning is lacking. Therefore, identification of the key teacher behaviors that raise student performance is a priority (Stroet et al., 2013, 2015a; Hagger and Hardcastle, 2014; Hagger and Chatzisarantis, 2015).

In this article, we begin by describing teaching quality and some of its related concepts. Then, we review researchers’ proposals of teaching quality dimensions within SDT. Next, we discuss the benefits of following SDT tenets in the classroom, as well as how engagement might mediate the relationship between teaching quality and academic achievement.

## Literature Review

### Teaching Quality

For the past 40 years, researchers using several frameworks have focused on the characteristics and practices of teachers who appear to be successful in their teaching (Kunter et al., 2013; Wagner et al., 2013). Unfortunately, researchers often use different terms for similar constructs and the same term for different ideas (Marsh et al., 2003; Seaton et al., 2014). For instance, we can use a number of terms to talk about classroom processes related with students learning: *Teaching quality* (Allen et al., 2011; Fauth et al., 2014), *quality of teaching* (Hattie, 2009) *teaching effectiveness* (Marsh and Roche, 1997; Seidel and Shavelson, 2007), *instructional quality* (Lipowsky et al., 2009; Rjosk et al., 2014), *teaching style* (Cai et al., 2002; Wentzel, 2002), and *instructional style* (Jang et al., 2010). Moreover, because teachers’ instructional practices refer to variables within the class level (Wagner et al., 2013), other terms used in the literature are *classroom quality* (Hamre et al., 2014), *classroom environment* (Day et al., 2015; McLean and Connor, 2015), and *classroom management* (Arens et al., 2015).

Within SDT, by “teaching quality” we refer to the specific teacher behaviors that supports the student needs of autonomy (feelings of self-determination and not being controlled), competence (feeling efficient and confident in the interactions with the social context), and relatedness (feeling connected and backed up by important others). Several authors have explored the different dimensions of teaching quality (See Ten Cate et al., 2011; Stroet et al., 2013; Liu et al., 2016a). In **Table 1** we present a summary of different dimension. In the next sections, we explained them in greater detail.

**Table 1:** Teaching quality dimensions and examples.

Dimension	Example
(A) Provide meaningful and explanatory rationales	Start a lesson by explaining how students might apply class contents in real life
(B) Nurture inner motivational resources	Use interesting and up-to-date examples
(C) Offer meaningful choices	Let students pick the topic in a class project
(D) Acknowledge negative feelings	Consider the anxiety that a student might feel when dealing with class activities
(E) Participation encouragement	Ask for the students’ opinions about a new topic
(F) Non-controlling language	Talk to students in a soft, informational tone using non-directive language
(G) Optimal challenge	Assign different class activities according to the students’ levels
(H) Focus on the process	Not only value the result, when revising student class activities
(I) Step-by-step instructions	Provide clear instructions and goals
(J) Class preparation	Spend time on activities and explanations before the class
(K) Positive feedback	Provide specific information about what is correct and what could be improve
(L) Caring	Pay attention to students’ feelings

#### Autonomy Support

*Autonomy* is the feeling of performing an activity self-determined, that is, from the highest level of reflection, or to put in other words, emanating from our self, without external pressures, and feelings of being the origin, agent, and cause of beginning and maintaining an activity (Stefanou et al., 2004; Domínguez et al., 2010). At school, students feel autonomous when they believe that school actions are not just an obligation but rather a mean to serve their interests (Wang and Eccles, 2013). When students feel forced to comply with school requirements, they feel controlled and not autonomous. Of course, at school, there are many situations and activities that make students feel controlled and not autonomous, but it is important to remark that this is not an “all or nothing” feeling, and it is in teachers’ hands to use different strategies to foster student autonomy.

In this sense, there are different teacher behaviors to support student autonomy in class: (A) *Provide meaningful and explanatory rationales*. Teachers ought to clarify why class contents and activities are important or useful (Guay et al., 2013; Stroet et al., 2013; Núñez et al., 2015). Explaining why schoolwork is important and relevant helps students to understand how it is in their interest (Assor et al., 2002). Specific teacher behaviors would be to start a lesson by explaining how students might apply class contents in real life or in other subjects, or by explaining how a specific class activity would help them. (B) *Nurture inner motivational resources:* Teachers could foster student autonomy by reinforcing student interests and developing student curiosity (Stroet et al., 2013; Reeve et al., 2014; Turner et al., 2014; Cheon and Reeve, 2015). A specific teacher behavior might be to explain class contents or frame class activities using interesting and up-to-date examples, or by asking curiosity-inducing questions. (C) *Offer meaningful choices:* Teachers could diminish student feelings of coercion by providing different options, allowing students to choose something closer to their interests (Núñez et al., 2012; Stroet et al., 2013; Vansteenkiste and Ryan, 2013). A specific teacher behavior could be to offer students the possibility to choose what exercises to do in an exam or to let them pick the topic in a class project. (D) A*cknowledge negative feelings:* To make students feel less coerced and controlled, teachers could pay attention and understand negative emotions that arise in class. For example, to consider the sadness, worry, or irritation that a student might feel when dealing with an exam or an activity that the student does not know how to solve (Assor et al., 2002; Taylor and Ntoumanis, 2007; Su and Reeve, 2010; Vansteenkiste et al., 2012). A specific teacher behavior could be to approach a student that is sobbing when doing an exam, and explain that it is common to feel anguish, but you know he or she is a hard worker. (E) *Participation encouragement:* Teachers should try to make students feel part of the class by requesting their opinions or encouraging them to participate in the learning process (Chatzisarantis et al., 2007; Roth et al., 2007; Gillet et al., 2011, 2012; Thapa et al., 2013). A specific teacher behavior might be to ask for the students’ opinions about a new topic or welcome student points of view. (F) *Non-controlling language:* Teachers ought to talk to students in a soft, informational tone using non-directive language and inviting forms instead of controlling forms (*you could* versus *you must*), and trying to focus on the didactics rather than on external pressures (Deci et al., 1989; Simons et al., 2005; Hagger et al., 2015).

#### Competence Support

*Competence* is the feeling of accomplishment and effectance when interacting with the environment (Ng et al., 2011) or, to put it another way, to know what it means and what it takes to be successful (Wang and Eccles, 2013). Children feel competent at school when they feel capable of achieving learning activities. Therefore, to foster student competence, teachers need to create structured, predictable, contingent, and consistent classrooms (Tessier et al., 2010).

More specifically, teachers need to provide: (A) *Optimal challenge*. Teachers must take into account the student’s level when assigning activities, so students can develop and exercise their capacities (Ryan and Powelson, 1991; Cheon and Reeve, 2015). A specific teacher behavior would be to assign different class activities according to the students’ levels. (B) *Focus on the process:* Teachers need to stress the importance of learning over to solving activities properly without internalizing its meaning and utility (Legault et al., 2006; Tessier et al., 2010; Kusurkar et al., 2011). A specific teacher behavior would be to take into account all of the procedure to solve a problem, and not only value the result, when revising student class activities or exams. Another teacher behavior would be to stress the importance of learning over exam results. (D) *Step-by-step instructions:* Teachers will provide clear goals and step-by-step instruction when assigning class activities; thus, students could know how to satisfy teacher expectations and achieve the selected academic outcomes (Skinner and Belmont, 1993; Jang et al., 2010; Vansteenkiste et al., 2012; Hospel and Galand, 2016). (D) C*lass preparation*: It is important for teachers to prepare the class well, explain precisely and clearly the class contents, use a good pedagogy during class, and structure the class session to avoid chaos and keep students, as much as possible, on task (Skinner and Belmont, 1993; Legault et al., 2006; Jang et al., 2010; de Naeghel et al., 2014). (E) *Positive feedback*: Guiding students to the desired goals and outcomes is a key teacher behavior optimally delivered using feedback (Thurlings et al., 2013; Hospel and Galand, 2016). According to Hattie and Timperley (2007), there are four different kinds of feedback, and not all are equally effective: *Feedback about the task* (FT), used to provide information about right or wrong answers or other specific issues; *feedback about the processing of the task* (FP), used to provide information about what strategies can be used to acquire a deeper learning; *feedback about self-regulation* (FR), used to provide information about student self-confidence or effort regulation; and *feedback about the student as a person* (FS), which includes praising a characteristic at the self and global level (e.g., “good boy”). This last kind of feedback is the least effective. The more specific the feedback is, the more powerful it is. Whereas FR and FP are useful to encourage a deep process and learning of the task, FT is powerful when the information provided leads to better strategies or bolsters self-regulation.

#### Relatedness Support

*Relatedness* is the need to build and maintain positive, meaningful, and lasting relationships (Baumeister and Leary, 1995). Students who feel related with their teacher, feel close, accepted, and backed up by their teacher (León and Liew, 2017). Teachers can foster student relatedness by demonstrating their trust and interest, by being available to them, or by paying attention to their feelings (Stroet et al., 2013). Students who do not feel related to teachers often disengage from class activities (Zee et al., 2013). Yet, when students feel close to and backed up by their teacher, it encourages them to think and learn (Baroody et al., 2014). Students who are more connected to teachers demonstrate positive trajectories of development in academic domains (Hamre and Pianta, 2010). If people are in an environment where they feel cared for and important, it increases the likelihood for the experience of learning out of pleasure and interest (Dietrich et al., 2015).

To sum up, many researchers have addressed the effects of teacher behavior following SDT tenets. However, they have focused not on a global approach but on specific items of each factor: autonomy support, competence support or relatedness support (Stroet et al., 2013). Some authors have focused on an observational basis, while others on self-repot. Observational studies within SDT have not predict strongly (or even not predicted) student academic functioning (Stroet et al., 2013, 2015b). Therefore, and bearing in mind that scales with specific items designed for students to evaluate teaching quality has shown evidence of reliability and validity (Bill and Melinda Gates Foundation, 2012; Wallace et al., 2016), in this study, we aim to develop a scale to assess student perceptions of the precise teacher behaviors that influence student performance.

### Behavioral Engagement as a Mediator between Teaching Quality and Math Performance

As mentioned previously, math performance has large implications on students’ lives (e.g., job opportunities, decision making, and self-esteem). To optimize student performance, researchers have explored the effect of teaching quality on student performance (Hattie, 2009). For instance, Riconscente (2014), in a year-long study with a sample of 9th- and 10th-grade students, observed that the students’ perception of teachers’ emphasis on class content interest, relevance, clarity, and caring predicted unique variance in student grades after accounting for demographics variables.

Nonetheless, research analyzing the mechanism by which teacher classroom behaviors affect student performance is still scarce (Ruzek et al., 2014). Therefore, we aim to shed some light on this topic. Skinner et al. (2009) propose that teacher behaviors affect student performance via motivation and engagement. In line with this proposition, Morin et al. (2014) differentiated between effects at both the class and individual level and concluded that students from 4th to 6th grade who perceived their classroom as challenging and their teacher as focused on mastery goals and interested in them, felt more competent, and this, in turn, bolstered their math achievement.

Many variables fall under the umbrella of motivation and engagement (Eccles, 2016); however, an indicator of behavioral engagement (BE) and a predictor of math achievement is effort regulation or effortful persistence, which can be understood as the perceptions of how much investment in time, energy, and work is dedicated to a task or a goal (Liew et al., 2011b). In another words, it is the students’ ability to exert effort and to persevere even when doing so is not easy or entertaining (Pintrich and de Groot, 1990). Depending on the theoretical framework, researchers have labeled this construct differently. One approach is the volitional framework (Corno and Kanfer, 1993; Corno, 2004). Experts under this framework would agree to explain it as the tendency to focus attention and direct effort toward goals despite distractions inside and outside schools (Chen, 2002; Pintrich, 2004). From a temperament perspective (Rothbart et al., 2003), the construct of “effortful control” refers to the capacity to regulate behavior and attention willingly (Liew et al., 2011a). Another similar construct is self-control or “grit,” the ability to consciously suppress prepotent responses in the service of a higher goal (Duckworth and Seligman, 2005; Duckworth and Steinberg, 2015). From a self-regulated learning perspective (Zimmerman, 2013), effort regulation can be defined as the student’s process to manage his or her behavior to achieve a goal; thus, self-regulated students are those who display appropriate levels of effort and persistence to attain their learning goals (Zimmerman, 1990; Zimmerman and Kitsantas, 2014). To sum up, we understand effort regulation as an indicator of engagement, which imply to keep on with school activities even if they are dull or uninteresting.

## The Purpose, Research Question, and Hypotheses of the Study

Within SDT research, much remains unknown about the specific and concrete teacher behaviors that foster student academic functioning (Stroet et al., 2013, 2015a; Hagger and Hardcastle, 2014; Hagger and Chatzisarantis, 2015). Therefore, our research question was: can we predict math achievement and engagement via teacher behaviors (teaching quality) asking students?

To answer this question, we depart from two ideas: (1) the individual student’s perception about their teacher behaviors might not be a precise indicator, however, if all students in one class perceive their teacher similarly, the average students perception might be a more precise indicator of their teacher behaviors; (2) according to Morin et al. (2014), student responses will vary because of individual perceptions (variance within classes), and because of shared perceptions among students in the same class (variance between classes). Therefore, our first goal was to develop and examine the psychometric properties of a scale for students to evaluate teacher behaviors according to the following dimensions: autonomy support, competence support and relatedness support. Specifically, we hypothesized that the scale would show a sound and robust multidimensional latent structure (Hypothesis 1a), the subscales would be positively associated with each other (Hypothesis 1b), and a significant amount of variance would be due to the group-level (Hypothesis 1c).

Our second goal was to test if the scale predicts student engagement and achievement. Drawing on the model of Skinner et al. (2009) and previous studies analyzing the effect of teaching quality on student engagement or motivation (e.g., Fauth et al., 2014; Morin et al., 2014; Longobardi et al., 2016), and the effect of the latter on achievement (Wang et al., 2015), it might be that in classes where the teacher provide meaningful and explanatory rationales, nurture inner motivational resources, offer meaningful choices, acknowledge negative feelings, encourage participation encouragement, use a non-controlling language, provide optimal challenge and step-by-step instructions, focus on the process, prepare the class, provide positive feedback and care about students, they would be more engaged in class, and are more persistent when dealing with school duties. Specifically, we predicted that at the class level, teaching quality would predict student effort regulation (Hypothesis 2a), and in classes where the students, as a whole, are more persistent on class activities, the average grade would be higher (Hypothesis 2b). Last, we predicted, at the individual level, students who persist and make more effort on school activities would achieve higher (Hypothesis 2c).

## Study 1

### Study 1 Method

#### Participants

Participants were 548 compulsory secondary students (52% males) with a mean age of 14.247 years (*SD* = 1.123). The students were grouped in 24 classrooms, with a mean number per class of 22.37 (minimum = 14; maximum = 30, *SD* = 3.70). Students were in grades 2 to 4 of secondary education, equivalent to 8th to 10th grade in the United States system (grade 8, *n* = 262, *M*_age_ = 13.48; grade 9, *n* = 124, *M*_age_ = 14.38; grade 10, *n* = 157, *M*_age_ = 15.43). The studied schools comprised a mix of urban and outlying rural public schools with students predominantly from middle-class families. Students attend 4 h of math lessons per week during the academic year. Students had time enough to know their teacher’s behavior in class, because the academic year had started 6 months before the assessment.

#### Procedure

Students provided informed consent to take part, and participation was strictly voluntary and confidential. Less than 1% declined to take part in the evaluation process. During the data collection, researchers administered the initial pool of items to all students in the classroom during March 2015, and provided them with instructions and clarifications if needed to complete the measures.

#### Measure

Building upon the SDT framework and previous scales designed to assess teaching quality, a group of research experts on SDT and math teachers designed a pool of 83 items (rated on a 7-point scale, 1 = *strongly disagree* to 7 = *strongly agree*) to cover specific and concrete teacher behaviors. Specifically, items were considered in relation to the following factors: (A) *Meaningful rationales provision*: The teacher explains why what students are learning is important or useful. (B) *Nurture inner motivational resources*: The teacher explains using interesting and up-to-date examples. (C) *Offer meaningful choices*: The teacher offers different options to students. (D) *Acknowledge negative feelings*: The teacher understands negative emotions that arise in class. (E) *Participation encouragement*: The teacher pushes students to take part in class. (F) *Controlling language*: The teacher talks to students using rigid and directive language. (G) *Optimal challenge*: The teacher takes into account the student’s level when assigning activities. (H) *Focus on the process*: The teacher stresses the importance of classwork and learning over marks. (I) *Step-by-step instructions*: The teacher explains precisely and systematically how to proceed with class activities. (J) *Classes preparation*: The teacher prepares and structures the classes well. (K) *Quick feedback*: The teacher provides feedback short after the behavior. (L) *Self-regulation feedback*: The teacher provides feedback about student self-confidence or effort. (M) *Specific feedback*: The teacher provides concrete and specific feedback. (N) *Caring*: the teacher looks after and pays attention to the students.

### Study 1 Results

To examine the factor structure, we performed a single-level confirmatory factor analysis (CFA) instead of a multilevel factor analysis because there were too many items for the number of classrooms and students to find a proper solution. Because all variables were ordered categorically, we used the Mean- and Variance-adjusted Weighted Least-Squares estimation method. The initial CFA with all items showed correlations higher than one between *Self-Regulation Feedback* and *Speed Feedback* (*r* = 1.055) and between *Providing Meaningful Rationales* and *Offering Choices* (*r* = 1.027), which might be an indicator of misfit. Therefore, to purify the scale (Hair et al., 2010, p. 666), we relied on information from parallel analysis (Horn, 1965; Hayton et al., 2004), exploratory structural equation modeling (ESEM; Asparouhov and Muthén, 2009), and Bayesian structural equation modeling (BSEM; Muthén and Asparouhov, 2012). As recommended by Asparouhov et al. (2015), to generate ideas about model modifications, first we accomplished a BSEM (cross-loadings priors with a distribution of mean 0 and variance 0.01). Next, we ran an ESEM with all of the items; however, the information provided was quite fuzzy, and we decided to divide the scale and analyze the data, exploring items in close factors based on the BSEM results and theoretical meanings.

We started by running a parallel analysis with the items from the factors: Meaningful Rationales Provision, Nurture Inner Motivational Resources, and Offer Meaningful Choices, concluding that a one-factor solution seemed adequate. This new factor assesses teacher emphasis on relevance, utility, and interests of class contents. Next, we removed items because of low loading values and for theoretical and practical reasons.

Concerning items from the factors: Optimal Challenge, Acknowledge Negative Feelings, Control Language, Provide Optimal Challenge, and Caring, we followed a similar procedure as described previously. We accomplished a parallel analysis, and observed that a five-factor solution was not the best, but there was not much difference in comparison with a lower number of factors. We decided to test a five-factor multilevel CFA and observed that the five-factor model showed an adequate fit: χ^2^(547, 1034) = 3026.221 (*p* = 0.00), RMSEA = 0.059, SRMR_*within*_ = 0.056, SRMR_*between*_ = 0.129, CFI = 0.962, and TLI = 0.958. Thus, we opted for this five-factor option; next, we removed items because of low loading values and for theoretical and practical reasons.

Finally, we ran a parallel analysis with items from the following factors: Focus on the Process, Class Preparation, Step-By-Step Instruction, and all Feedback items. We observed that three factors seem a good statistical and theoretical solution, and that items from Step-By-Step Instruction and Class tended to load on the same factor, something understandable, as both assess the teacher structure in the classroom. The next step was to remove items from this factor because of low loadings values and for theoretical and practical reasons.

Once we had the final 53 items and 9 factors, we ran a multilevel CFA (MCFA); the χ^2^ value and fit indexes were χ^2^(547, 2578) = 4583.151 (*p* = 0.00), RMSEA = 0.038, SRMR_*within*_ = 0.052, SRMR_*between*_ = 0.121, CFI = 0.980, and TLI = 0.979. Loadings at the individual level ranged from 0.431 to 0.798. With regard to correlations, at the within level, they ranged from 0.846 (Caring with Positive Feedback) to 0.261 (Controlling Language with Focus on the Process), and at the classroom level, they ranged from 1 (Optimal Challenge with Teaching for Relevance) to 0.755 (Controlling Language with Teaching for Relevance).

To examine reliability, instead of Cronbach’s alpha, we used McDonald’s Omega (McDonald, 1999) because the former requires factor loadings to be equal for all items (McNeish, 2017) and data to be continuous (Elosua and Zumbo, 2008). Further, McDonald’s Omega has shown evidence of better accuracy compared with Cronbach’s alpha (Revelle and Zinbarg, 2009). McDonalds’ values should be interpreted in a similar fashion as Cronbach’s alpha is: values above 0.70 to 0.80 are indicators of reliability. **Table 2** shows that McDonald’s Omega varied from 0.650 (Focus on the Process) to 0.893 (Positive Feedback).

**Table 2:** Descriptive statistics, intraclass correlation, reliability and correlations at the within and between level for all major variables.

	Variable	Items	*M*	*SD*	ICC	ICC2	ω	1	2	3	4	5	6	7	8	
1	Teaching for relevance	9	3.788	1.468	0.343	0.923	0.867		0.958	0.937	-0.711	1.000	0.976	0.841	0.927	0.946
2	Acknowledge negative feelings	6	3.622	1.562	0.415	0.942	0.818	0.649		0.953	-0.825	0.958	0.886	0.789	0.948	0.975
3	Participation encouragement	5	4.319	1.528	0.361	0.928	0.754	0.715	0.658		-0.824	0.960	0.879	0.905	0.932	0.939
4	Controlling language	5	5.473	1.416	0.294	0.905	0.739	-0.386	-0.371	-0.366		0.755	0.780	0.762	0.886	0.757
5	Optimal challenge	4	4.165	1.594	0.309	0.911	0.747	0.619	0.624	0.632	0.384		0.966	0.860	0.961	0.948
6	Focus on the process	4	4.740	1.444	0.013	0.225	0.650	0.280	0.351	0.372	0.162	0.324		0.992	0.900	0.829
7	Class structure	5	5.323	1.451	0.180	0.834	0.865	0.511	0.415	0.501	0.451	0.518	0.262		0.867	0.766
8	Positive feedback	8	4.623	1.557	0.285	0.901	0.893	0.729	0.686	0.740	0.467	0.681	0.381	0.610		0.930
9	Caring	7	3.955	1.562	0.351	0.925	0.867	0.713	0.724	0.710	0.425	0.703	0.332	0.572	0.813	

*ICC, intraclass correlation; ω, McDonald’s omega; Lower diagonal triangle, within level correlations; Upper diagonal triangle, between level correlations; Upper diagonal triangle, correlations at the class level*.

To examine the average agreement between students, or the proportion of the total variance at the group level, we estimated intraclass correlation (ICC1). Values close to 1 indicate that all of the variance is due to the class, whereas values close to 0, indicate that the variability is due to the subjects and not to the group. ICC1 varied between 0.415 for Acknowledge negative feelings and 0.013 for Focus on the Process (**Table 2**). Finally, to test the reliability as a group construct, we estimated ICC2. Values close to one are evidence that students in the same classroom share the same feelings or thoughts, whereas values close to one indicate that the construct assessed is independent among students (Morin et al., 2014). ICC2 varied from 0.942 (Acknowledge Negative Feelings) to 0.225 (Focus on the process).

### Study 1 Discussion

The first aim of the study was to develop, purify, and examine the psychometric properties of a scale to teacher behaviors according to SDT tenets asking students. We hypothesized that the scale would show a sound and robust multidimensional latent structure, the subscales would be positively associated with each other, and a significant amount of variance would be due to group-level variance.

We developed a pool of 83 items designed to assess 14 factors. After a purifying process, we had a 53 items and 9 factor scale. The factors are teaching for relevance, acknowledge negative feelings, participation encouragement, controlling language, optimal challenge, focus on the process, class structure, positive feedback, and caring. The reduction from 14 to 9 factors was achieved because items designed to measure different feedback factors were merged in one factor. Similarly, the factors meaningful rationales provision, nurture inner motivational resources, and offer meaningful choices, were merged in a unique factor called teaching for relevance. Likewise, items from step-by-step instruction and class preparation were merged into one factor. These results are in line with previous research (Assor et al., 2002; Wang and Eccles, 2013), suggesting that students feel autonomous when they do not feel coerced. Thus, if teachers offer different options but none satisfy their interests or are not useful for students, they would still not feel autonomous. It is not just offering choices or options, but also opening up the range of meaningful possibilities to cover student interests and priorities what matters. Therefore, it seems likely that the factor to assess is teaching for relevance, meaning that the teacher relies on useful and interesting class content and activities, to provide different options to reach the majority of students. We also merged in one-factor (class structure) items designed to tap two factors: Step-by-step instruction and class preparation. Step-by-step instructions refer to if teacher explains activities and contents clearly, and class preparation if the teacher knows the class content and organize the class well. Thus, although we hypothesized that students would understand them as two different factors, students seem to perceive it as one factor. It might be that if teachers prepare the classes, they also prepare the activities.

With regard to the hypothesis of observing a sound and robust multidimensional latent structure, reliabilities were adequate, and results of the MCFA revealed that the data fit a nine-factor model, with strong loadings on the intended factors, and correlations between factors. At the individual level, correlations values were moderate, but at the within level, higher values were observed, which is in line with previous studies assessing teaching quality: for instance, Fauth et al. (2014) observed a correlation of 0.89 between cognitive activation and supportive climate, and Morin et al. (2014) observed a correlation of 0.92 between mastery goal structure and challenge. Fast et al. (2010) and Morin et al. (2014) posit that these high correlations might be because factors fall under a common denominator: the teaching style, so it makes sense that student perception of teacher behavior represents a higher-order factor.

We expected that a meaningful part of the variance would be due to the class level variance, and in line with previous studies assessing teacher or class behaviors (Fauth et al., 2014; Decristan et al., 2015). We observed that the scale seems to capture the group nature of a class evaluation. To sum up, these results provided support for sound psychometric properties, supporting the factorial validity of the scale. However, we need evidence that the purified scale fits the data in another sample and predicts student engagement and achievement.

## Study 2

### Study 2 Method

#### Participants

Participants were 1555 compulsory secondary students (51% females; mean age = 15.30 years, *SD* = 1.12) grouped in 82 classrooms from nine schools, in grades 2 to 4 of secondary education, equivalent to 8th to 10th grade in the United States system (Grade 8, *n* = 588, *M*_age_ = 13.94; Grade 9, *n* = 484, *M*_age_ = 15.01; Grade 10, *n* = 483, *M*_age_ = 16.19). The studied schools comprised a mix of urban and outlying rural public schools with students predominantly from middle-class families. Students attend to 4 h of math lessons per week during the academic year. They had time enough to know their teacher’s behavior in class, because the academic year had started 6 months before the assessment.

#### Procedure

We followed a similar procedure as in the previous study. Students provided informed consent to participate, and partaking was strictly voluntary and confidential. Less than 1% declined to take part in the evaluation process. During the data collection, in May 2015, researchers administered the instruments to students in their classrooms and provided students with instructions and clarifications if needed to complete the measures. At the end of the school year in June, we obtained the student final course grades in mathematics from school records. To maintain anonymity, the school provided records without the name, just the class and the birthdate of each student, which we later linked with the questionnaire of each student. However, in the same class, 5 times three students were born on the same day, and 26 times two students were born on the same day; therefore, we could not match grades with questionnaires for these 67 students.

#### Measures

To analyze scale reliability, we computed McDonald’s Omega. To estimate how much variance was due to group-level variance, we estimated ICC1. To examine the reliability of the measure as a group indicator we calculated ICC2. Finally, to test the factor structure, we ran a MCFA.

##### Teaching quality

We used the 53-item scale described in the previous study. The scale assesses nine factors: (A) *Teaching for relevance*: the teacher uses useful and interesting class contents and activities. (B) *Acknowledge negative feelings*: The teacher understands negative emotions arisen in class. (C) *Participation encouragement*: The teacher pushes students to take part in class, by asking questions or soliciting students’ opinions. (D) *Controlling language*: The teacher talks to student in rigid and directive language. (E) *Optimal challenge*: The teacher takes into account student level when assigning activities. (F) *Focus on the process*: The teacher stresses the importance of classwork and learning over marks. (G) *Classes structure*: The teacher prepares and structures the classes and activities well. (H) *Positive feedback*: The feedback provided is quick, positive, and specific. (I) *Caring*: The teacher looks after and pays attention to students. Reliability ranged from 0.919 (Positive Feedback) to 0.804 (Focus on the Process). ICC1 ranged from 0.545 (Teaching for Relevance) to 0.342 (Focus on the Process). ICC2 ranged from 0.957 (Teaching for Relevance) to 0.905 (Focus on the Process) (**Table 3**). Finally, concerning the MCFA, the χ^2^ value and fit indexes were χ^2^(1524, 2578) = 19843.661 (*p* < 0.001), RMSEA = 0.067, SRMR_*within*_ = 0.046, SRMR_*between*_ = 0.054, CFI = 0.966, and TLI = 0.964. All items are listed in the Supplemental Material.

**Table 3:** Descriptive statistics, intraclass correlation, reliability and correlations at the within and between level for all major variables.

	Variable	*M*	*SD*	ICC1	ICC2	ω	1	2	3	4	5	6	7	8	9	10	11
1	Teaching for relevance	4.033	1.683	0.545	0.957	0.889		0.946	0.960	-0.495	0.931	0.917	0.874	0.944	0.852	0.409	0.232
2	Acknowledge negative feelings	3.449	1.772	0.467	0.942	0.892	0.684		0.961	-0.531	0.966	0.965	0.809	0.964	0.911	0.443	0.284
3	Participation encouragement	4.436	1.655	0.385	0.921	0.828	0.618	0.598		-0.563	0.935	0.939	0.852	0.958	0.846	0.405	0.181
4	Controlling language	2.732	1.575	0.416	0.930	0.827	-0.147	-0.105	-0.123		-0.469	-0.518	-0.453	-0.602	-0.600	-0.335	0.015
5	Optimal challenge	4.123	1.736	0.472	0.943	0.871	0.532	0.551	0.633	-0.171		0.942	0.847	0.942	0.842	0.434	0.209
6	Focus on the process	4.447	1.732	0.342	0.905	0.804	0.470	0.468	0.544	-0.140	0.569		0.804	0.949	0.846	0.487	0.361
7	Class structure	5.181	1.668	0.400	0.926	0.908	0.519	0.436	0.566	-0.221	0.548	0.571		0.855	0.728	0.327	0.121
8	Positive feedback	4.534	1.706	0.387	0.921	0.919	0.669	0.661	0.721	-0.224	0.687	0.648	0.652		0.923	0.369	0.200
9	Caring	3.907	1.663	0.423	0.931	0.902	0.660	0.750	0.670	-0.138	0.610	0.526	0.521	0.739		0.333	0.242
10	Effort regulation	4.636	1.378	0.161	0.782	0.728	0.199	0.191	0.220	-0.119	0.288	0.209	0.264	0.285	0.294		0.481
11	Math grades	5.217	2.212	0.084	–	–	0.099	0.113	0.113	-0.091	0.257	0.199	0.158	0.183	0.167	0.467	

*ICC, intraclass correlation; ω, McDonald’s omega; Lower diagonal triangle: within level correlations; Upper diagonal triangle: between level correlations; Upper diagonal triangle: correlations at the class level*.

##### Effort regulation

Student *effort regulation* was assessed using four items from the effort regulation subscale of the Motivated Strategies for Learning Questionnaire (Pintrich et al., 1993) on a 7-point scale (1 = *strongly disagree* to 7 = *strongly agree*). Sample items included “When work is difficult, I either give up or study only the easy parts.” All items on this measure have demonstrated adequate reliability (ω = 0.74) in prior research (León et al., 2015) and in the present study (ω = 0.716). ICC1 was 0.168, and ICC2 was 0.783. With regard to the MCFA, residual correlation between two of the four items that were worded in a similar way was allowed; the χ^2^ value and fit indexes were χ^2^(1524, 1) = 6.471 (*p* = 0.039), RMSEA = 0.038, SRMR_*within*_ = 0.010, SRMR_*between*_ = 0.013, CFI = 0.999, and TLI = 0.994.

##### Math grades

Student math performance was indexed by student final course grades in mathematics, which we obtained from the official high school records. Unlike in the United States or United Kingdom, where it is common to assess students using standardized test, in Spain we rely more on school grades assigned by teachers, because there is not such a variety of standardized tests. Teachers have to assign grades using rubrics implemented by the Government based on student knowledge, skills and work in class and at home.

These grades have real-world significance on student academic standing and progress in grade school (Thorsen and Cliffordson, 2012; Simões and Alarcão, 2014; Sánchez-Pérez et al., 2015). Actually, in Spain, students choose different tracks and even different universities based on their high school grades. Grades were coded as 1 being the lowest and 10 being the highest possible mark. Teachers give an average score based on student’s skills, knowledge, and homework, as required by Spanish curriculum.

#### Data Analysis

Descriptive analyses and correlations between major variables, at the within and between level, were conducted. Next, we tested the second hypothesis by running a multilevel structural equation model (MSEM), where at the individual level, effort regulation predicted math performance, and at the class level teaching quality predicted math performance via effort regulation. To test if effort regulation mediated the effect of teaching quality on math performance, we added, in a nested MSEM, a direct effect from teaching quality to math performance; we can conclude that, if this direct effect is not significantly different from zero and the fit of the two-nested model is not different, effort regulation is a mediational variable. We handled missing data using the full information maximum-likelihood method with the Mean-adjusted Weighted Least-Squares estimator (Asparouhov and Muthén, 2010).

### Study 2 Results

#### Descriptive Analysis and Correlations at the within and the between Level

Descriptive statistics (means and standard deviations) and correlation for all major variables are displayed in **Table 3**. The means varied between 2.732 (Controlling Language) and 5.217 (math grades), and standard deviations between 1.378 (Effort Regulation) and 2.212 (math grades). With regard to correlations, at the within level, they ranged from 0.687 (Optimal Challenge with Positive Feedback) to -0.091 (Controlling Language with math grades), and at the between level, they ranged from 0.965 (Acknowledge Negative Feelings and Optimal Challenge) to 0.015 (Controlling Language with math grades).

#### Multilevel Model

**FIGURE 1:**
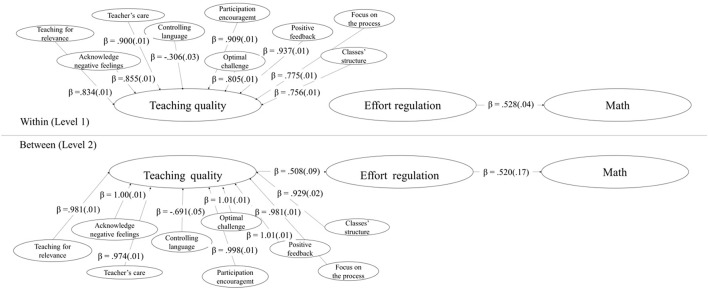
Multilevel structural equation model. All effects are significant (*p* < 0.001). Standard errors between parentheses.

The χ^2^ test and fit indexes for the MSEM χ^2^(1504, 3170) = 23506.247 (*p* = 0.00), RMSEA = 0.065, SRMR_*within*_ = 0.052, SRMR_*between*_ = 0.087, CFI = 0.962, and TLI = 0.961. In **Figure 1**, we can see, that, at the within level, all Teaching Quality factors loaded on a higher-order factor. Effort Regulation predicted math grades (β = 0.528; SE = 0.036; *p* < 0.001), explaining 28% of its variance. Whereas at the between level, every Teaching Quality factors loaded on its factor, and Teaching Quality predicted Effort Regulation (β = 0.508; SE = 0.097; *p* < 0.001), and this, in terms, math grades (β = 0.520; SE = 0.171; *p* < 0.001); explaining 26% and 27% of its variance, respectively.

With regard to the mediational effect at the between level of Effort Regulation, in the relationship between Teaching Quality and math grades, we compared the above mentioned MSEM with a MSEM with an additional path from math grades to Teaching Quality. The χ^2^ test and fit indexes for this MSEM were χ^2^(1527, 3169) = 23549.585 (*p* = 0.00), RMSEA = 0.065, SRMR_*within*_ = 0.052, SRMR_*between*_ = 0.087, CFI = 0.962, and TLI = 0.961. The χ^2^ test (adjusting for the correction factor) comparing both models was not significant: Δχ^2^(1527, 1) = 2.921 (*p* > 0.05), and the direct effect from Teaching Quality to math grades was not different from zero (β = -0.076; SE = 0.146; *p* = 0.66). Therefore, we can conclude that, at the between level, Effort Regulation mediates the relationship between Teaching Quality and math achievement.

### Study 2 Discussion

This study provides support for the hypotheses tested. At the between level, teaching quality was found to be a predictor of effort regulation (Hypothesis 2a): higher grades were observed in classes where students, as a whole, displayed more effort regulation (Hypothesis 2b). At the within level, students who showed more effort regulation on school activities achieved higher grades (Hypothesis 2c). Therefore, Study 2 provides more support of the scale developed to assess teaching quality.

#### Teaching Quality and Behavioral Engagement

With regard to Hypothesis 2a, we observed that when the students, as a whole, perceived that their teacher provided quality teaching, more BE was displayed. This in line with the model of Skinner et al. (2009), who propose that teaching quality might predict student academic functioning (e.g., motivation, engagement, and achievement). In this research, we have conceptualized “quality teaching” as when the teacher conducts lessons with useful and interesting class contents and activities (teaching for relevance); understands negative emotions that arise in class (acknowledge negative feelings); pushes students to take part in class (participation encouragement); talks to student in non-controlling and attuned language (controlling language); takes into account students’ levels when assigning activities (optimal challenge); stresses the importance of classwork and learning over marks (focus on the process); prepares and structures the classes and activities well (class structure); provides feedback that is quick, positive, and specific (positive feedback); and looks after and pays attention to students (caring). It seems that specific teacher behaviors, such as calming down students when they are nervous doing an exam or bearing in mind the students’ levels when assigning class activities, promote students’ persistence when studying math. Effort regulation is necessary for students to achieve many school activities and to pay attention in class and perform when there are some more appealing activities to do (watching TV or playing videogames). Thus, educators who provide a quality teaching, as conceptualized in this research, are providing students with prerequisites for persisting on school activities.

#### Behavioral Engagement and Math Grades

In line with hypotheses 2b and 2c, the multilevel model showed that BE had predictive power for grades. At the individual level, these results, in addition to those from others researchers (Duckworth et al., 2012; Hofer et al., 2012), suggest that students get better grades if they gain the capacity to persist studying even when they find it dull or prefer to do something else. We observed similar results at the class level, where higher grades are observed in classes where the students, as a whole, display more effort regulation. Thus, we agree with Veronneau et al. (2014) that educators who want students to achieve as high as possible should pay attention to students and try to foster effort regulation in them.

#### Indirect Effect of Behavioral Engagement between Teaching Quality and Math Grades

Our results indicate that effort regulation, as an indicator of BE, is one mechanism that mediates the link between teaching quality and math performance. These results are in line with the model of Skinner et al. (2009). Other researchers have also focused on the effect of teaching quality on math performance. For example, Kunter et al. (2013), assessing mainly teachers instead of students, observed that pedagogical content knowledge and teacher enthusiasm predicted math grades via the use of applied math problems and classroom disruption and discipline. Morin et al. (2014) also focused on teaching quality and math performance. They observed that self-efficacy mediated the relationship between those two variables. Our study adds to the previous studies a stronger prediction of math grades. Whereas Kunter et al. (2013) explained 13% of math achievement variance, and Morin et al. (2014) reported and effect size of 0.15, we explained 27% of the variance at the between and 28% at the within level. Moreover, in this research, we assessed multiple specific teacher behaviors, which researchers or practitioners could use to design useful interventions to promote student achievement.

## General Discussion

Educators face the challenge of engaging students to learn and achieve during middle and high school math lessons. Teaching quality during class have important influence on student functioning. However, within SDT, the precise teacher behaviors that lead to optimal functioning are not well defined. Therefore, in the first study, we proposed an instrument to assess specific and concrete teacher behaviors, and in the second study, we identified a mechanism by which these teacher behaviors predict math achievement.

In study 1, we developed a scale to assess teaching quality with nine factors to capture specific and concrete teacher behaviors. The developed scale provided evidence of reliability and factorial validity. However, we needed evidence that the scale fit the nine-factor structure in another sample, and that it predicts student engagement and achievement.

Study 2 builds on existing literature that underscores the importance of teaching quality as key predictors of educational attainment (Skinner et al., 2009; Fauth et al., 2014). We observed that effort regulation mediated the relationship between teaching quality and grades. It is important to highlight that it could be that the educators with a positive quality teaching might assign higher grades to students. However, as shown in the results section, the direct effect of teaching quality on math grades, controlling for effort regulation, was not different from zero because effort regulation is the linking variable between teaching quality and math achievement. To put it different, with the precaution of not making causal claims, it seems that teaching quality promotes students’ effort regulation, and this, in turn, promotes math achievement. Thus, it seems as it is not that teachers with a better teaching quality assign higher grades, but, that these teachers move students to put more effort on their school activities, which, in turn, leads to higher grades.

### Strengths and Limitations

Our study included a number of strengths: We conducted two separate studies to analyze scale psychometric properties, and in the second study, we included two waves of data. In the first one, we assessed teacher behavior and student effort regulation, whereas in the second, we collected math grades 1 month later.

Student ratings of teacher behavior assess the typical teacher performance along the course, and have been proposed as an accepted method to evaluate teaching methods (Wagner et al., 2013; OECD, 2014; Wallace et al., 2016). Usually, teacher observation assesses just one or more days of class behaviors, where the teacher might strive to teach as “good as possible,” but we are aware that this observational information might help to grasp a bigger picture of the classroom. Therefore, future studies could gather observational information of teaching quality based on the nine factors proposed in this study.

One limitation regarding study 1 is the limited sample size. This fact, among the high correlations between different factors, precluded us of reaching convergence in the MCFA. Subsequently, we aimed to purify the scale accomplishing an ESEM, unfortunately, results were fuzzy, that is, we did not have information about the optimal number of factors nor about the relationship between items and factors. Therefore, we proceeded to a step-by-step procedure based on the BSEM results and theoretical information. Although this procedure might look arbitrary, we believe that the proposed scale has theoretical foundations and evidence of reliability and validity to warrant its use.

It is also important to stress that this research has been conducted in Spain. In this country, and other European countries, grades are of ultimate importance: students based on their academic grades choose track and, later on, University. Thus, in Spain grades is a variable of ultimate importance for student life. Researchers in other countries, such as United States or United Kingdom, could assess the impact of the scale on other variables such as the SAT, ACT or GCSE.

Finally, the effects of teaching quality on student learning can be diverse. As pointed out by Seidel and Shavelson (2007), some teacher behaviors might have short-term effects (e.g., interest and enthusiasm), whereas others might have longer effects (e.g., motives to study and study strategies). Therefore, it might be interesting to study the effect on different variables, beyond effort regulation and math grades. In a similar fashion, although the study was designed under the SDT umbrella we did not assess key variables such as autonomy, competence or relatedness, because our goal was not to predict these three psychological variables, but to predict behavioral indicators such as student BE and achievement. However, we believe that it could be interesting for future research to test the relationship between the teaching quality factors and autonomy, competence or relatedness. Finally, we believe that it could be interesting to test the effect of school variables such as percentage of students receiving free and reduced-price meals or school climate (Konold, 2016) on teaching quality.

## Conclusion

In this study, we aimed to shed some light in the discovery of new path to optimize students’ math achievement. Therefore, we focused on a variable amenable to intervention: teaching quality. We provide a reliable and valid instrument for students to assess specific and concrete teachers’ behaviors during class, which we grouped under the label teaching quality. The findings of this study have implications for practitioners and researchers. The former could use the developed scale to assess their teaching quality, while researchers could design interventions based on the scale items to promote student persistence and effort on school duties, which in turn, bolsters student achievement.

## Ethics Statement

This study was carried out with written informed consent from all subjects. All subjects gave written informed consent in accordance with the Declaration of Helsinki. The protocol was approved by the University of Las Palmas de Gran Canaria.

## Author Contributions

Conception or design of the work: JL. Data collection: JL, EM-G. Data analysis and interpretation: JL, EM-G. Writing the manuscript: JL, EM-G. Edit the manuscript: JN. Final approval of the version to be published: JL, EM-G, JN.

## Conflict of Interest Statement

The authors declare that the research was conducted in the absence of any commercial or financial relationships that could be construed as a potential conflict of interest.
